# A Population of Deletion Mutants and an Integrated Mapping and Exome-seq Pipeline for Gene Discovery in Maize

**DOI:** 10.1534/g3.116.030528

**Published:** 2016-06-01

**Authors:** Shangang Jia, Aixia Li, Kyla Morton, Penny Avoles-Kianian, Shahryar F. Kianian, Chi Zhang, David Holding

**Affiliations:** * Department of Agronomy and Horticulture, Center for Plant Science Innovation, Beadle Center for Biotechnology, University of Nebraska, Lincoln, Nebraska 68588; † USDA-ARS Cereal Disease Laboratory, University of Minnesota, St. Paul, Minnesota 55108; ‡ School of Biological Sciences, Center for Plant Science and Innovation, Beadle Center for Biotechnology, University of Nebraska, Lincoln, Nebraska 68588

**Keywords:** maize functional genomics, BSR-seq, exome-seq, opaque, causative mutation

## Abstract

To better understand maize endosperm filling and maturation, we used γ-irradiation of the B73 maize reference line to generate mutants with opaque endosperm and reduced kernel fill phenotypes, and created a population of 1788 lines including 39 Mo17 × F2s showing stable, segregating, and viable kernel phenotypes. For molecular characterization of the mutants, we developed a novel functional genomics platform that combined bulked segregant RNA and exome sequencing (BSREx-seq) to map causative mutations and identify candidate genes within mapping intervals. To exemplify the utility of the mutants and provide proof-of-concept for the bioinformatics platform, we present detailed characterization of line 937, an opaque mutant harboring a 6203 bp in-frame deletion covering six exons within the *Opaque-1* gene. In addition, we describe mutant line 146 which contains a 4.8 kb intragene deletion within the *Sugary-1* gene and line 916 in which an 8.6 kb deletion knocks out a *Cyclin A2* gene. The publically available algorithm developed in this work improves the identification of causative deletions and its corresponding gaps within mapping peaks. This study demonstrates the utility of γ-irradiation for forward genetics in large nondense genomes such as maize since deletions often affect single genes. Furthermore, we show how this classical mutagenesis method becomes applicable for functional genomics when combined with state-of-the-art genomics tools.

There are a variety of existing resources for forward and reverse genetics in maize (McCarty and Meeley 2009; Settles 2009). These are based on EMS (ethyl methane sulphonate), transposons, and radiation-induced physical alterations to DNA. EMS mutagenesis creates mostly point mutations and often results in knock-down alleles that are useful for studying gene function. With the availability of the complete maize genome and the ability to map genes through techniques such as BSR-seq (bulked segregant RNA-seq) (Liu *et al.* 2012, 2013), map-based cloning of EMS mutants in maize is feasible. The Targeting Induced Local Lesions in Genomes (TILLING) approach (McCallum *et al.* 2000) has been developed for high-throughput identification of EMS point mutations in a variety of plant species including maize (Till *et al.* 2004). Transposons, which create a high frequency of knock-out alleles, have been used in a variety of forward mutagenesis schemes and have led to various reverse genetics resources such as the Mutator-based Uniform Mu project (McCarty *et al.* 2005; Settles *et al.* 2007) and an *Ac*/*Ds* based system (Vollbrecht *et al.* 2010). Proving successful cloning through additional alleles is often a bottleneck in the completion of mutant cloning procedures and so existing and new mutant collections, such as the one described here, increase options for both forward and potentially reverse genetics in maize.

Though transposon insertions are useful for creating nulls, mutations can also be leaky. Likewise, EMS mutations often result in knock-down alleles. Physical deletion of genes, as achieved through irradiation, is one way to remove this ambiguity. Radiation has also been used for inducing physical deletions of genes and this has been shown to be a viable system for PCR-based reverse genetics in *Arabidopsi*s (Li *et al.* 2001). Although deletion mutants were obtained for over 80% of targeted loci from an *Arabidopsis* population of more than 50,000 lines, such near saturation based on random deletions would be a major challenge in plants with much larger genomes such as maize. However, deletion mutagenesis has potential as a tool for investigating biological processes of interest, such as embryo and endosperm development and other general aspects of vegetative and reproductive plant development. In bringing together genomic sequencing of the maize exome and BSR-seq, a bulked segregant mapping approach based on quantitative typing of SNPs derived from RNA-seq reads (Liu *et al.* 2012, 2013), we are able to appraise the frequency and size of deletions.

The potential of γ-irradiation for reverse genetics has been investigated (Wu *et al.* 2005; Sato *et al.* 2006). Although the mutagenesis rate is lower than with EMS mutagenesis, γ-irradiation results in a higher proportion of knock-out mutations (Sato *et al.* 2006). The dosage of radiation must be carefully optimized to maximize the mutagenesis rate while allowing an acceptable level of viability of the M1 plants. Low and moderate doses cause a relatively high proportion of useful mutants with normal yield (Dumanovi *et al.* 1969) while high doses induce a higher proportion of large deletions containing genes essential for viability (Naito *et al.* 2005). In an *Arabidopsis* irradiation experiment, the majority of large deletions were not transmitted from the M1 to the M2 generation and were assumed to result in deletion of genes required for seed germination, gamete development, or other aspects of reproductive development (Naito *et al.* 2005). However, in large genomes with lower gene density, the tolerance for large deletion may be higher. The chromosomal alterations in *Arabidopsis* included point mutations, translocations, insertions, small 1 to 4 bp deletions and large deletions ranging in size from kilobases to several megabases. In a rice mutagenesis experiment, the rate of mutation was ∼1 mutation per 6.2 Mb (Sato *et al.* 2006). With current genomics technologies, radiation mutagenesis has greater potential for studying gene and genome function. To this end, a large fast neutron population in soybean was created for functional genomics of agriculturally important traits (Bolon *et al.* 2011).

We have demonstrated the utility of γ-radiation for functional genomics in maize by knocking out the 27 kD γ-zein gene, the major modifier of the *opaque-2* phenotype in quality protein maize (Yuan *et al.* 2014). Here, we describe the use of γ-radiation to create a collection of novel heritable seed mutants in the B73 maize reference genetic background. We prioritized opaque kernel and reduced kernel size mutants that did not substantially affect kernel viability or vegetative development. The other main objectives were to appraise this mutagenesis method for functional genomics by investigating the occurrence of kilobase scale deletions that are most likely to affect single genes as well as to develop a method to rapidly and affordably map and identify causative deletions. To identify causative mutations in the induced mutants, we built a complete pipeline, bulked segregant RNA and exome-capture sequencing (BSREx-seq), by combining BSR-seq (Liu *et al.* 2012) and exome-seq (exome sequencing), plus genomic PCR and RT-PCR verification. We generated a new allele of the *o1* mutant (line 937) and used it as an example to prove that our pipeline can successfully map the gene and identify the causative deletion within the mapping interval. Significantly, the deletion in mutant 937 is entirely contained within one gene thus supporting this method for maize functional genomics. We describe examples of mapping causative mutations in other mutants some of which correspond to very strong deletion candidates. We also describe a new allele of the *sugary1* mutation which also harbors an intragenic deletion that we identified with exome-seq alone. We developed an improved BSR-seq analysis pipeline, which was optimized to display causative deletions as their corresponding gaps within the linkage peak. Our analysis highlights the potential for this mutant resource for identifying genes involved in various aspects of seed and whole plant development using the BSREx-seq pipeline.

## Materials and Methods

### Radiation dosage testing and γ-radiation mutagenesis

Freshly harvested B73 maize seeds were equilibrated to 13% moisture as previously described (Hossain *et al.* 2004) and treated in batches of ∼50 seeds with various dosages of γ-radiation (15 to 35 Gy). After irradiation, the seeds were immediately planted in the greenhouse in potting soil. Plant survival was determined as the proportion of seedlings compared to nonirradiated control seeds 1 month after sowing. Radiation dosage was set at 25 Gy based on a 75% survival rate and was used for full scale mutagenesis of 4000 seeds. These M0 seeds were planted in the field at the University of Nebraska East Campus farm in summer 2012.

### Propagation, screening for kernel phenotypes, and creation of mapping populations

**Figure 1:**
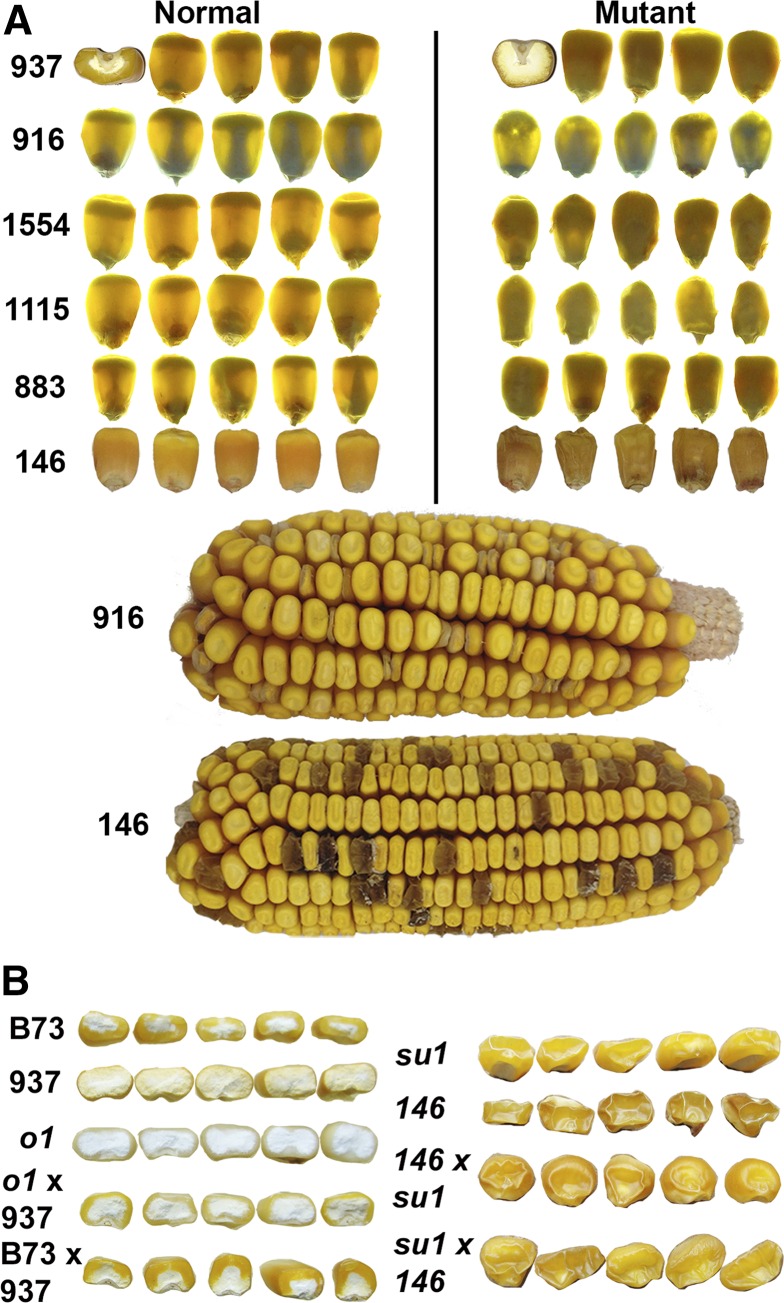
Opaque and reduced-fill kernel phenotypes. (A) Opaque phenotypes were shown on light box. Normal phenotype and mutant phenotype are shown for kernels from B73 × Mo17 F2 segregating ears. (B) 937/*o1* and 146/*su1* allelism tests.

M1 ears were harvested from M0 plants. Recessive mutant phenotypes are not visible in the M1 ears due to the already physically separate ontogeny of the tassel and ear founder cells in the embryonic maize shoot apical meristem (Johri and Coe 1983; Poethig *et al.* 1986). However, potentially dominant phenotypes were noted. All M1 ears were propagated to the M2 families (10−20 ears per family) in 2013 and 2014 and families were scored for segregating phenotypes including opaque, small kernel, small kernel opaque, narrow, thin, rough surface, partially empty pericarp, shrunken, white, fully empty pericarp (EP), viviparous (VP) (precocious germination), severe defective kernel (DEK). Six seeds from lines carrying a possible mutant phenotype, with the exception of EP, VP, and DEK classes which are invariably lethal, were tested for germination (Table 1). Plants which germinated and survived past the seedling stage, thus having reduced likelihood of nonseed specific pleiotropic effects, were propagated to adulthood in the greenhouse and self-pollinated and their pollen used to generate an F1 outcross to Mo17. In some cases M2 ears were not obtained but the more reliably obtained F1 was propagated to the F2 (5−6 plants) in all cases. Where a mutant phenotype was penetrant in the B73/Mo17 hybrid background and not specific to the B73 genetic background, this was displayed as a segregating phenotype in all F2 ears which were large, healthy due to hybrid vigor, and thus easy to reliably screen (Supplemental Material, Table S1). A small fraction of confirmed mutants (Figure 1A) were selected for BSR-seq based on the F2 phenotype and the ability to germinate M2 seeds for the exome-capture experiment.

**Table 1:** Viability, heritability tests, and production of B73 F2 mapping populations

M2 Families in 2013–14	Putative M2 Families	Seed Lethal	Seedling Lethal	Viable	Mo17 × B73 F1s Obtained	F2 lines Showing Segregating Seed Phenotypes
1788	266	97	36	133	93	39

### BSR-seq

**Figure 2:**
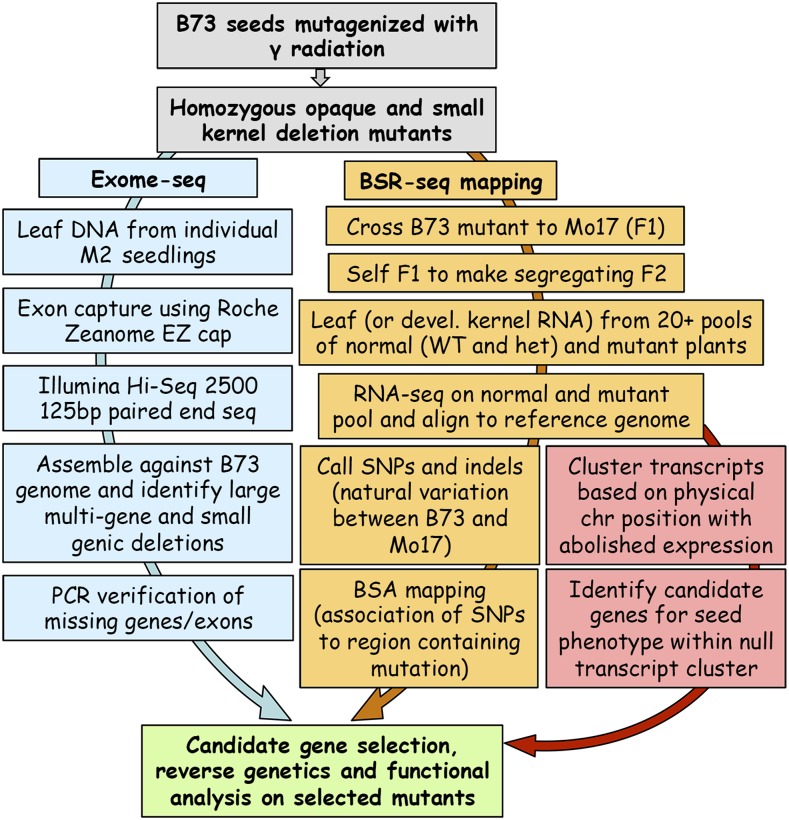
BSREx-seq workflow.

Approximately 30 normal-type (wild-type and hemizygous) seeds and 30 mutant seeds from selected segregating F2 ears were planted in small pots and grown in the greenhouse for 2 wk. Approximately 5 cm^2^ of leaf tissue was sampled from each plant and pooled to produce normal-type (N) and mutant-type (M) pools (Figure 2). The plant number in one BSR-seq pool varied from 21 to 30, and the pools with less than 30 plants resulted from less than 100% germination (Table S2). Total RNA was extracted, DNase1 treated, and repurified using a previously described method (Holding *et al.* 2011). Libraries were prepared from RNA-seq by the University of Minnesota Genomics Center using TruSeq RNA v2 kits and samples were bar-coded so that each HiSequation 2500 lane had 10 samples (five mutants each with a normal-type and mutant-type pool). Inserts of about 200 bp were selected by gel size. Sequencing was conducted on a 50 bp paired-end run on the HiSequation 2500 using rapid chemistry.

### Exome sequencing

Genomic DNA was extracted from leaf tissue from B73 wild-type and M3 single mutant 14-d-old leaf material according to a previous method (Holding *et al.* 2008). High-quality DNA was prepared by using Qiagen DNA clean-up kit, after its concentration and integrity were checked using a NanoDrop and agarose gel analysis. Purified DNA was sent out for exon capture and HiSeq sequencing at the University of Minnesota Genomics Center. The Maize_ISU_RNG_Zeaome_EZ_HX1 SeqCap EZ kit (Roche) enriched ∼110 Mb exon regions (http://xtalks.com/Exome-Sequencing-for-Crops.ashx), which covered 39,621 genes and 190,641 exons in B73 annotation (version: AGPv3.25), and was used to purify mutant samples with four bar-coded samples per column. After the exon capture step, the samples were further pooled so that each HiSequation 2500 lane contained the exome fraction from eight mutants. Sequencing was conducted on a 125 bp paired-end run on the HiSequation 2500 using v4 chemistry.

### SNP/indel calling for BSR-seq and exome-seq

All raw short reads were preprocessed by trimming off low-quality nucleotides on individual reads and/or removing low-quality whole reads with Trimmomatic v0.32 (Bolger *et al.* 2014). The minimum average quality score was set at 28 and the minimum length was 45 bp for BSR-seq and 100 bp for exome-seq data, respectively. The preprocessed high-quality reads (Table S2) were then mapped against the B73 reference genome (AGPv3.25) using Bowtie2 v2.2.4 (Langmead and Salzberg 2012) with default parameters. VarScan v2.3.7 (Koboldt *et al.* 2012) was employed for SNP/indel calling from the pair-wise pileup files, the aligned base-pair information at one chromosomal position listed in one line, generated by Samtools v0.1.19 (Li 2011) after merging, sorting, and indexing the mapped reads. The minimum read depth for a SNP/indel call is five. During SNP/indel calling, a given position was considered as a SNP/indel if there were only two alleles and the frequency of each allele was more than 20% of the depth at this position.

### Expressed gene counts and assembly

Preprocessed BSR-seq reads were also aligned to the B73 reference with TopHat (Trapnell *et al.* 2009), and numbers of reads mapped were counted using HTSeq v0.6.1 (Anders *et al.* 2015) based on the B73 annotation (AGPv3.25). For pair-wise comparisons, differentially expressed genes were identified by using DEseq (Anders and Huber 2010) for *P*-value calculation. Transcriptome assembly was implemented by Cufflinks (Trapnell *et al.* 2012) after mapping reads to the reference genome with TopHat, and assembled transcripts in normal and mutant pools were compared to each other with Cuffcompare.

### Linkage analysis for BSR-seq

In the BSR-seq analysis, F2 plants of B73 mutant × Mo17 are expected to consist of three genotypes, AA, Aa, and aa, among which AA and Aa plants show the normal phenotypes, but aa plants show the mutant phenotypes. The goal of BSR-seq is to identify the genomic area that is closely linked to aa alleles. Therefore, in this area, two alleles in the mutant pool are homozygous and both are from B73. During BSR-seq analysis, the sequence variants between Mo17 and B73 worked as markers to identify the linkage regions which show the linkage disequilibrium in the whole genome. After steps of BSR-seq read mapping against the B73 reference genome and SNP/indel calling for all reads from both normal and mutant pools, the SNPs/indels with more than five reads in both normal and mutant pools, only two alleles, and either allele having >20% depth were kept. One allele is from B73 and the other from Mo17. For a chromosomal position having this kind of SNP/indel, if its Mo17 allele, *i.e.*, the one from the Mo17 origin rather than the B73 one has a fraction from 50% (Aa) to 100% (AA) in the normal pool, and it is homozygous in the mutant pool, *i.e.* a pure B73 allele without any sequence variants, then this position was considered as having a positive SNP/indel. For all positive SNPs/indels (Table S2), a sliding-window of 100 kb with a step of 10 kb was used to calculate the number of positive SNPs/indels per window, where a high number indicates a linkage peak, closely linked to a causative mutation. The numbers of positive SNPs/indels in sliding-windows were displayed with R and Bioconductor package “ggbio” (Yin *et al.* 2012) in the whole genome. In the linkage peak region, high numbers of positive SNPs/indels in sliding-windows, the causative deletion would be identified with combining the exome-seq data analysis. At the same time, a large read-free region in either normal or mutant pool, possibly due to the sequencing bias, gene expression regulation, or γ-radiation, was not considered for positive SNPs/indel discovery, but would be considered as a candidate for DNA deletion if its expression was uniquely found in the normal pool underneath the linkage peak.

### Deleted exon identification using exome-seq

Deleted exons, which are mutations most likely caused by γ-radiation, were identified from exome-seq analysis. Preprocessed exome-seq reads were aligned to the B73 reference genome with BWA mem (Li and Durbin 2010), a mapping tool designed for long reads, and depth for each chromosomal position was achieved by Samtools. The accumulated depth (*D*) for a given exon was the sum of sequencing depth of all positions in this exon, and the normalized depth of an exon was calculated by the equation: (10^9^ × *D*)/(*LD*_*a*_), where *D*_*a*_ is the total accumulated depth of the whole genome and *L* is the exon length. An exon was considered as a deleted one if the normalized accumulated depth for this exon is more than 30 in B73 but 0 in the mutants.

### Filtering out the common mutations from background

Due to the nonidentical nature of different seed stocks of the same inbred in use by maize researchers, the B73 background of all mutants generated in this population has common genetic variation from the B73 reference genome which is shared in all mutants. This variation causes false positives and will be identified by searching common mutations among two or more mutants. Since the chance of two mutants having γ-radiation-caused deletions with identical coordinates is essentially 0, such common deletions among mutants cannot result from the mutagenesis and cannot be causative. Indeed, our BSR-seq analysis showed that no causative candidate mutations were found in the same genome locus in any pair of mutants. To identify these mutations from the background, all discovered mutations, including deleted exons, and SNPs/indels, were scanned for the occurrence in all mutants. The mutations with occurrences of two or more were discarded, and the remaining ones consisted of the mutant-unique variations (likely not causative) and mutations induced by γ-radiation which are potentially causative. The unique deletions were double-checked in Integrative Genomics Viewer (IGV, version 2.3) (Thorvaldsdottir *et al.* 2013) to exclude the bias caused by sequencing and exon capture steps.

Searching for the causative deletion in each mutant was conducted using BSR-seq alone, exome-seq alone, and BSR-seq and exome-seq together. The Perl script developed for BSREx-seq data analysis is freely downloadable on our website (http://sysbio.unl.edu/BSREx/).

### Zein analysis and western analysis

Total protein as well as zein and nonzein fractions were extracted from mature and developing endosperm according to the previous method (Wallace *et al.* 1990). Western analysis of the OPAQUE-1 protein was conducted according to the previous method (Guo *et al.* 2012). The O1 antibody was a gift from Rentao Song and was used at 1/500 dilution.

### Genomic PCR and RT-PCR verification

For causative gene candidates, we wrote a Perl script to extract the sequences from B73 genome reference, and used Primer3 to design batch primers with melting temperatures of around 60°, PCR product size range of 50 bp to ∼500 bp (the optimized 200 bp), and primer size range of around 25 bp. We also called Blastn program to estimate the primers’ specificity across the whole genome, to make sure the primers will produce unique PCR product with leaf genomic DNA or complementary DNA (cDNA) as template. A step-moving strategy with a window size of 500 bp and a step size of 200 bp was used to generate the best primers inside genes or exons. All primers in this study are included in Table S3. The Perl script (GPPD, Genomic PCR Primer Design) is deposited on our website (http://sysbio.unl.edu/BSREx/).

### Data availability

Seeds from all lines are available on request. The BSR-Seq and exome-seq data have been deposited in the Short Reads Archive (SRA) database (http://www.ncbi.nlm.nih.gov/sra) of National Center for Biotechnology Information (NCBI) with the accession number of SRP067758. 

## Results

### Identification of maize kernel mutants in a B73 deletion population

Four thousand mutagenized B73 maize kernels (M0) were propagated to the M1 ears. After loss of plants due to failed germination, lethal somatic effects, and male or female sterility, 1788 variably filled M1 ears were recovered. During the summers of 2013 and 2014, one row (10−20 plants) of each family were propagated to the M2 ears which were then screened for segregating seed mutant phenotypes. Although it was anticipated that many putative mutant phenotypes would be environmental and not heritable, 266 families showing potential genetically caused variation were tested (Table 1). All mutant classes, except DEK, EP, and VP, were tested for seed and seedling viability and those that survived to the reproductive stage were self-pollinated to assess heritability and outcrossed to Mo17 to create an F1 (Table S1). The F1 was then self-pollinated to make an F2 mapping population. In some cases, the F2 was the first visible confirmation of the heritability of the phenotype where the M2 plant self-pollination did not produce an M3 ear. Five mutants, including lines 1039-*o2* (opaque-2 control mutant), 937 (full size opaque kernel), 1115 (small opaque kernel), 1554 (small, thin kernel with variable opacity), and 883 (full size opaque kernel), for which both BSR-seq and exome-seq data were generated, were selected for demonstrating the BSREx-seq pipeline (Table S2). Mutant 146, which exhibited a shrunken kernel phenotype, was included in the exome-seq experiment although an F2 mapping population was not yet available for BSR-seq. Mutant 916 (small, thin partial-filled opaque kernel) was only subjected to BSR-seq and not exome-seq. Kernel phenotypes are shown in Figure 1A. In the mutants tested, the F2 ears visibly segregated 1/4 mutant, and chi-square tests showed that the ratios were in accordance with mendelian inheritance (1:3, Table S4). Normal (wild and hemizygous/heterozygous types) and mutant seeds from segregating F2 ears were planted in the greenhouse, and a single young leaf from each of 30 plants was collected and pooled for RNA extraction. Meanwhile, the nonhybrid M2/M3 seeds were planted as well as wild-type B73, for leaf DNA extraction in exome-seq (Figure 2).

### BSREx-seq identified the causative o2 deletion in the control mutant 1039-o2

To test the limits of the power of exome-seq and BSR-seq to identify a causative deletion, we used a test line 1039-*o2* created by crossing a random B73 M0 plant (line 1039) to W64A*opaque2* (*o2*), self-pollinating the M1/F1 and selecting for homozygous *o2* seeds in the segregating M2/F2 ears. Since this mutant is a genomic mosaic of both B73 and W64A, the resulting presence/absence variation from pure B73 presents a rigorous test of both BSR-seq and exome-seq. The resulting B73/W64a hybrid had a full size opaque phenotype (Figure S1A) caused by the homozygous *o2* mutation. 1039-*o2* mutant exhibited typical *o2* opaque phenotype and zein and nonzein profiles characteristic of *o2* (Figure S1B).

During BSR-seq data analysis for 1039-*o2*, about 455,000 high-confidence SNPs/indels from all 10 chromosomes were retrieved (Table S2), and their density was shown as orange bars in Figure S1C. After using sliding-window analysis, the peaks of positive SNPs/indels, indicating the location of linkage disequilibrium, were plotted against their physical coordinates in the B73 reference genome (red bars in Figure S1C). In 1039-*o2*, positive SNPs/indels intensively appeared on chromosome 7. The highest linkage peak was identified as around 12.5 Mb, indicating linkage disequilibrium with causative mutation in the *o2* gene (GRMZM2G015534, chr7: 10,798,621–10,800,828).

After exome-seq reads in 1039-*o2* were mapped to the B73 reference genome, the read abundance in each exon was analyzed, and many read-free exons, mainly due to the variation between B73 and W64A, were detected and named as “absent exons” (blue bars in Figure S1C). Exome-seq analysis for 1039-*o2* showed that the genomic region with the highest linkage peak does not have any absent exons, while they are abundant in the neighboring regions surrounding the peak. The number of absent exons and other genomic variations are much greater than in other pure B73 mutants and these genomic variations evenly distributed across the genome, suggesting the mosaic genome context. This is consistent with the significant differences expected between the W64A genome and the B73 reference genome, and this genomic variation may have been made more pronounced by the fact that the exome-capture kit does not include W64A sequence and the exome-captured reads for the W64A genome could not always be well aligned to the B73 reference genome.Despite the challenge for identifying causative deletions with many absent exons in a non-B73 genome using exome-seq, in principle most of which are false positives, we identified the known 25 bp causative deletion inside the *o2* gene in mutant 1039-*o2* (Figure S1, C and D). The NCBI BLAST search showed the *o2* gene sequence in exome-seq is the same as the reference sequence (AJ491298.1) of o2-T allele (Lazzari *et al.* 2002; Gavazzi *et al.* 2007) in W64Ao2, and our 1039-*o2* mutant was homozygous for the 25 bp deletion.

### BSR-seq identified strong candidates for causative deletions in mutants 916 and 1554

**Figure 3:**
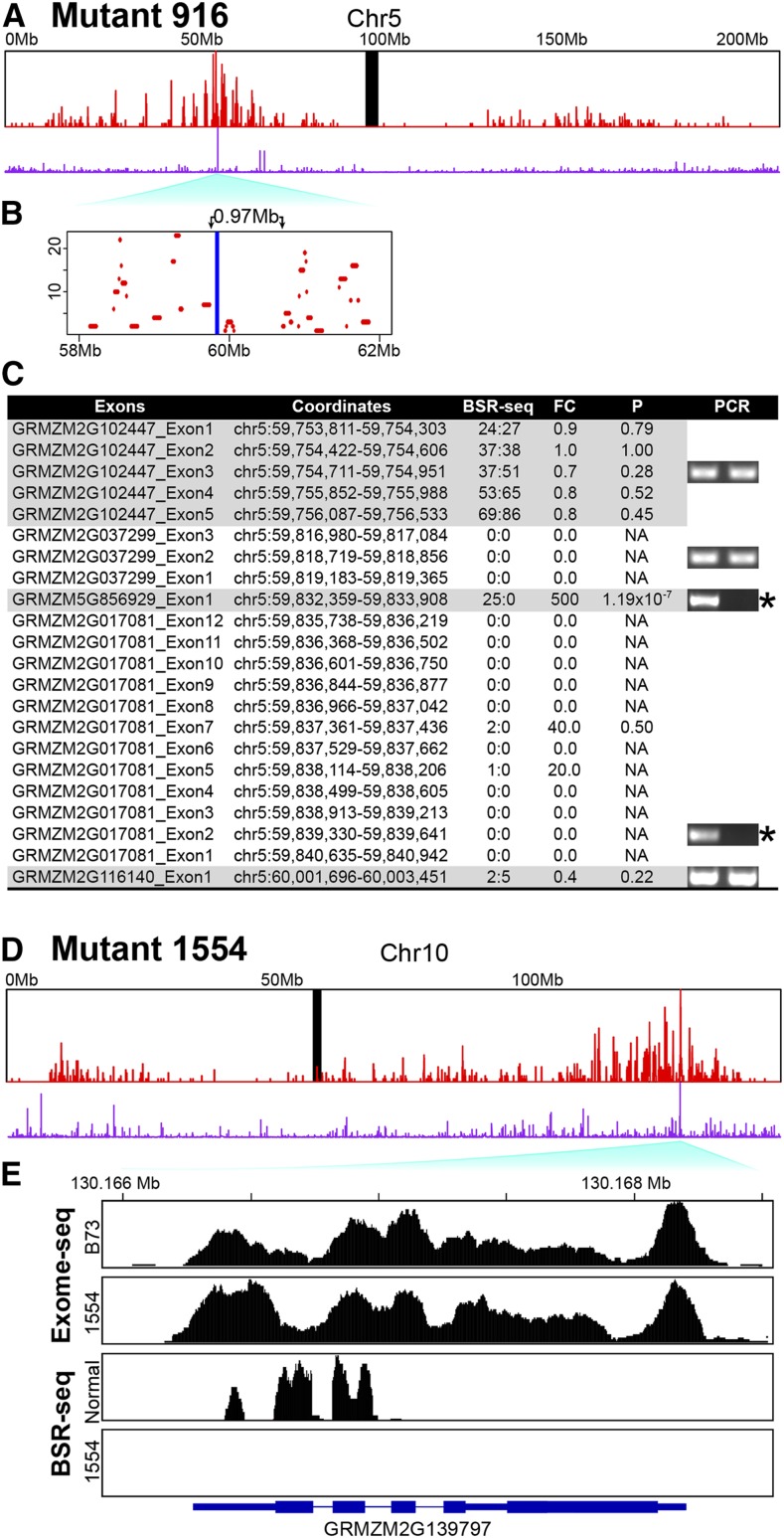
BSR-seq linkage analysis identified causative gene candidates in mutants 916 and 1554 using expression ratio. Expression difference overlapped with linkage peaks on chromosome 5 in mutant 916 (A) and on chromosome 10 in mutant 1554 (D). Red bars: numbers of positive SNP/indel in sliding-window of 100 kb with a step of 10 kb; purple bars: fold-change of gene expression values between mutant pool and normal pool based on BSR-seq reads; black bar: centromere. Y axis scale for positive SNPs/indels ranges from 0 to 23 and 31 in mutants 916 and 1554, respectively. (B) An enlargement of the highest linkage peak region, 58–62 Mb, was shown in mutant 916, in which a gap (∼0.97 Mb in blue) contains the causative deletion. (C) PCR verification of a causative deletion containing two genes on chromosome 5 in mutant 916. Normalized numbers of read counts of normal type (N) and mutant (M) were shown. Expression fold-changes (FC) defined as N/(M + 0.05) and *P*-values calculated by R package DESeq, were used to search for deleted exons. The verified deletions are noted with an asterisk. (E) The mapping coverage of the gene GRMZM2G139797 in exome-seq and BSR-seq are visualized.

We tested whether BSR-seq alone can identify the causative deletion for mutant 916. The linkage peak is at the genomic position 59.3 Mb of chromosome 5, in which the causative deletion is expected to be located (Figure 3A and Figure S2A). Since γ-radiation mutagenesis can induce large deletions (Yuan *et al.* 2014), which could span longer than one gene, we searched for genes with high read abundances in the normal pool (wild type and hemizygous) and no reads in the mutant pool. Gene GRMZM5G856929 (5:59,832,359–59,833,908) showed a very significant difference in read abundance between normal and mutant 916. Without exome-seq, extending forward and backward across the chromosome 5 to a ∼250 kb region surrounding this gene, five genes were treated as deletion candidates for further genomic PCR verification, among which two end genes on the chromosome 5 were shown not deleted due to being expressed in the mutant and the others were deletion candidates. Finally, genomic PCR showed that two genes (GRMZM5G856929 and GRMZM2G017081) were confirmed deleted, and the other three neighboring genes were not (Figure 3C). The deletion region was estimated to be of ∼8.6 kb from 59,832,359 to 59,840,942 on chromosome 5, within a linkage gap of 0.97 Mb (Figure 3B). It was noted that GRMZM2G017081 is annotated as *Cyclin-A2*. Cyclins, which mainly consist of A-, B-, and D-type, are the activating units of CDKs, and formation of cyclin–CDK complexes is essential for kinase activity in cell cycle regulation. It was suggested that the *proline responding1* gene mutation in an opaque mutant can result in down-regulation of cyclins, which caused cell cycle arrest at the transition from G1 to S phase, suppression of cell proliferation, and finally the seedling lethality phenotype (Wang *et al.* 2014). A similar phenotype of small and thin partial-filled opaque kernels was seen in mutant 916. Dante *et al.* (2014) and Sabelli *et al.* (2014) described core cell cycle factors that play important roles in the regulation of the cell division cycle during endosperm development (Dante *et al.* 2014; Sabelli *et al.* 2014). It is likely that the *Cyclin-A2* gene is the causative gene mutation in 916, with its embryo specific expression (Figure S3), while the other gene GRMZM5G856929 was shown to have leaf-specific expression based on the expression results of MaizeGDB website (http://www.maizegdb.org/).

In mutant 1554, one gene GRMZM2G139797, which had significantly differential read abundance between normal and mutant in the F2 generation, fell within the BSR-seq linkage peak region on chromosome 10 (Figure 3D and Figure S2B). This suggested that, at a distance of 72 kb from the BSR-seq linkage peak, the gene GRMZM2G139797 with gene name ZmCLO2b (Table 2), which has a large number of reads in the normal pool and no reads in the mutant pool, is a strong candidate for the causative gene. This gene encodes a caleosin family member, and likely plays a role in seed oil body formation (Hu *et al.* 2014) which would be expected to directly affect embryo development more than endosperm development. Thus, characterizing reduced seed fill mutants also can shed light on processes involved in carbon partitioning and communication between embryo and endosperm during development. It was noted that exome-seq analysis found no gene deleted in the highest linkage peak region, and ZmCLO2b itself is not deleted (Figure 3E). This may suggest a causative mutation in the intergenic regions close to this gene, although three deleted exons and one small deletion were found in other regions (Table S2).

**Table 2:** Causative deletion candidates identified by BSREx-seq

	BSR-seq*^a^*	Exome-seq*^b^*	Causative Deletion Candidates	Description
1039_*o2*	chr7:12.5 Mb	chr7:10,800,211-10,800,237	GRMZM2G015534 (chr7:10,798,621-10,801,375)	Chr7: 25 bp deletion in *OPAQUE-2* gene
916	chr5:59.3 Mb	NA	GRMZM2G017081 (chr5:59,835,738-59,840,942)*^c^*	Chr5: Cyclin-A2 in a ∼8.6 kb deletion
1554	chr10:130.2 Mb	None overlapped*^d^*	GRMZM2G139797 (chr10: 130,166,275-130,168,20)*^c^*	Chr10: ZmCLO2b, a caleosin family member
146	NA	chr4:41,397,178-41,401,983	GRMZM2G138060 (chr4:41,396,390-41,405,179)	Chr4: 10 exons deleted in *SUGARY-1* gene, ∼4.8 kb
1115	chr3:137.7 Mb	chr3:125,369,536-127,035,245	>86 exons in 26 genes	Chr3: 1.7-Mb deletion
937	chr4:174.9 Mb	chr4:176,912,920-176,919,560	GRMZM2G449909 (chr4:176,900,008-176,926,797)	Chr4: Six exons deleted in *OPAQUE-1* gene, 6203 bp
883	chr8:13.2 Mb	None overlapped*^d^*	Possible regulatory elements	Chr8: beyond coding regions

a In BSR-seq, linkage peak was plotted across the whole genome, and its genome coordinate was shown (chrosome#:coordinate, Mb).

b In exome-seq, the deletion overlapped with the linkage peak was used as causative deletion candidate.

c Mutants 916 and 1554 were with a causative gene candidate based on BSR-seq gene expression difference.

d Exome-seq analysis showed that no gene/exon showed a deletion in the linkage peak region of mutants 883 and 1554. And a deletion in the regulatory region might be beyond coding regions.

### Exome-seq alone identified the causative deletion in mutant 146

Exome-seq was applied to mutants 146, 1039-*o2*, 1554, 1115, 937, and 883 (not 916). For mutant 146, after exome-capture, the HiSeq sequencing results showed that more than 189,000 out of a total of 190,641 Ensemble maize annotated exons had at least five reads. Since more than 99.2% of exons were captured, it was possible to search for the gene/exon absence. The deleted exons and other mutations in the mutants were identified by comparing mutants and wild-type B73. The detected variation consisted of γ-radiation introduced mutations and individual noninduced variations, and the noninduced variations were identified by searching common ones (occurrence ≥2) among all mutants, including 105 deleted exons, 21,368 small indels, and 225,632 SNPs. The removal of this common genetic variation significantly reduced the identified causative mutation candidates.

Exome-seq analysis of mutant 146 identified one deletion (∼4.8 kb) on chromosome 4 (Figure S4A), containing 10 exons (exons #2–#11) in GRMZM2G138060 (Figure S4B), the *SUGARY-1* gene (*SU1*), plus 11 small indels. The deletion region (4804 bp) was estimated to extend from 41,397,178 to 41,401,983, where the left break point was located inside the exon #2 body (41,397,089–41,397,274) and the right break point broke the intron between exons #11 and #12. It indicated that nine whole exons (exon #3–#11) were deleted in the *SU1* gene, and part of exon #2 was lost.

The 79 kD protein encoded by the *SU1* gene functions as an isoamylase-type starch-debranching enzyme for starch biosynthesis and accumulates in endosperm (Rahman *et al.* 1998; Dinges *et al.* 2001). The *su1* mutant has increased sucrose concentration, decreased concentration of amylopectin, and a shrunken kernel phenotype (James *et al.* 1995). The same kernel phenotype was found in the mutant 146 (Figure 1). To test whether the identified deletion in *SU1* gene was causative, we did reciprocal crosses between mutant 146 and a reference *su1* mutant (407D), and confirmed that their offspring had the shrunken kernel phenotype (Figure 1B) and so they do not complement each other and are thus allelic.

### Exome-seq identified a large deletion under the BSR-mapping peak in mutants 1115 and 937

The combination of BSR-seq and exome-seq were used to identify causative deletions in mutants 1115 and 937 and this illustrates that deletions can be large and include many genes in the case of 1115 or relatively small and affect only a single gene in the case of 937. In mutant 1115, deleted exons identified by exome-seq data scatter on three chromosomes (3, 5, and 7), while the BSR-seq linkage peak resides only on chromosome 3 (Figure S5A). The linkage peaks on chromosome 3 are distributed widely, and the highest peak is at the genomic site 137,660,000 with a distance of ∼10 Mb to a large deletion, which suggests that this large deletion interrupted the random chromosomal recombination and resulted in a wide linkage peak. Therefore, the combination of exome-seq and BSR-seq analysis results revealed a large deletion (∼1.7 Mb) on chromosome 3 between 125,369,536 and 127,036,462 (Figure S5, B and C), which covers 86 exons from 26 genes (Table S5). Although dissecting which gene causes the phenotype and whether or not the phenotype results from the loss of more than one gene is not straightforward, one can infer the causative deletion is on chromosome 3.

**Figure 4:**
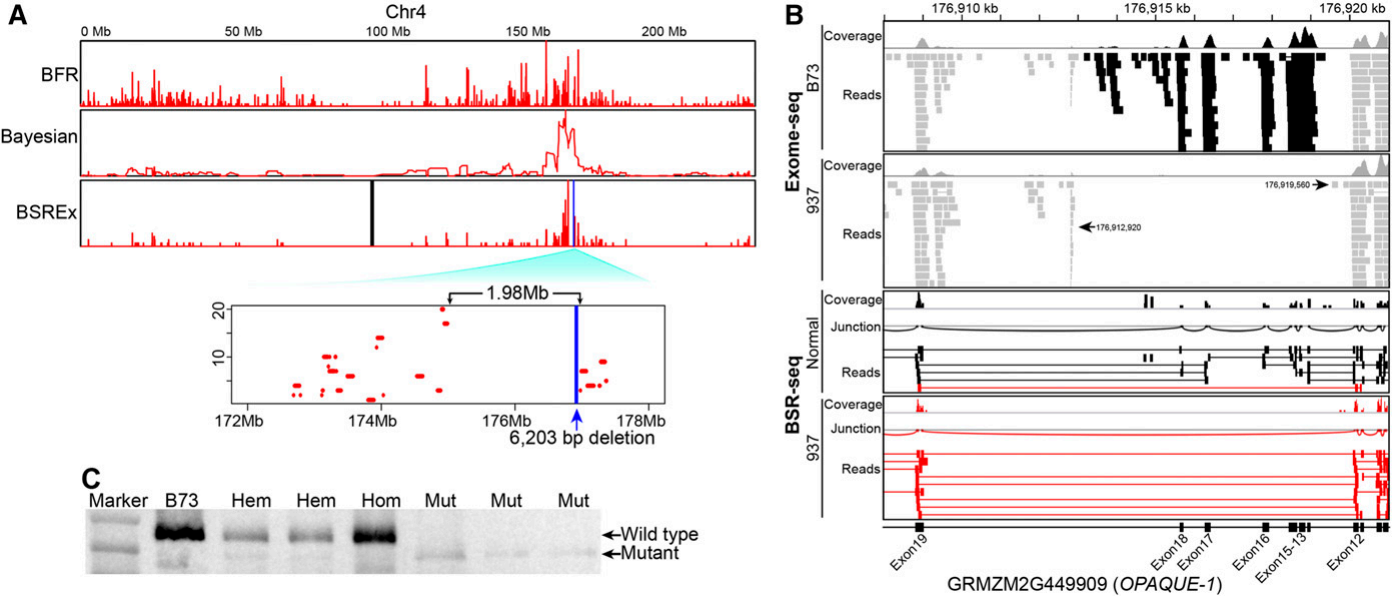
Identification of causative deletion candidate in mutant 937 by combining BSR-seq and exome-seq. (A) The current BSR-seq analysis and two other BSR-seq methods, Bayesian (Liu *et al.* 2012) and BFR (Ramirez-Gonzalez *et al.* 2015), all mapped the causative gene to chromosome 4 in mutant 937. Enlargement of a sharp linkage peak (red), 172–178 Mb, and a gap (∼1.98 Mb) covering the 6203 bp deletion (blue) spanning six exons of *O1* gene inside the linkage peak were shown. Black bar: centromere. Y axis scale for positive SNPs/indels ranges from 0 to 20. (B) The alignments of exome-seq reads (gray) around the causative deletion, including six exons (#13–#18, in black), are shown in the coverage and read windows. The alignment of BSR-seq reads showed two transcript variants, one for normal type (black), and the other (red) for mutant 937 without six exons of *o1* gene. (C) Western blot shows two protein variants with different lengths: wild-type and mutant truncated proteins. Hem, hemizygous-type kernel with both wild-type and mutant truncated proteins; Hom, homozygous wild-type kernel with only wild-type proteins; Mut, mutant-type kernel with only mutant truncated proteins.

Unlike the large deletion in mutant 1115, a short intragene deletion was found in mutant 937. A sharp linkage peak on chromosome 4 in mutant 937 suggested a strong linkage of the opaque phenotype to the causative gene at genomic position around 174.9 Mb (Figure 4A and Figure S2C), where the highest linkage peak was shown. Exome-seq analysis identified a deletion underneath this linkage peak. The deletion resides within the *OPAQUE-1* (*O1*) gene (GRMZM2G449909), and results in the loss of six exons (#13–#18). According to the BSR-seq reads, *O1* was expressed in leaf of both normal-type and mutant 937 (raw reads count ratio, 407:381), but no reads were mapped to the six deleted exons in mutant 937. The deletion break points on two ends of the deletion were estimated at 176,912,920 inside the intron of exon #12–#13 and 176,919,560 inside the intron of exon #18–#19, resulting in an ∼6.6 kb deletion (Figure 4B). Cufflinks was used to assemble transcripts in wild type and 937 and confirmed that the six exons were deleted in the expressed *o1* gene (Table S6), and found two wild-type transcripts, GRMZM2G449909_T1 and GRMZM2G449909_T2. Furthermore, TopHat alignment results showed that both transcript variants were found in normal F2 plants (wild type and hemizygous), while only the mutant transcript without the six exons was present in the mutant (Figure 4B).

### Comparison of BSR-seq analysis algorithms in mutant 937

Our BSR-seq analysis pipeline was optimized to produce a sharp mapping linkage peak close to the causative deletion, and had the ability to detect a gap in the BSR-seq linkage peak caused by the deletion. The original BSR-seq analysis algorithm developed in maize by Liu *et al.* (2012) used the empirical Bayesian approach to calculate the probability of meeting a 1:2 ratio of mutant to wild-type alleles in the nonmutant pool, in a 50-SNPs window (Liu *et al.* 2012). We optimized the strategy, and directly calculated the positive SNP number in a physical 100 kb window with a moving step of 10 kb. Our BSR-seq analysis algorithm was compared to the previous Bayesian (Liu *et al.* 2012) and bulk frequency ratio (BFR) (Trick *et al.* 2012; Ramirez-Gonzalez *et al.* 2015) methods for mutant 937, to evaluate their performance. All three BSR-seq analysis methods showed linkage peaks for the causative deletion on chromosome 4 (Figure 4A), but the BFR method resulted in suboptimal results with increased noise and many false positives (BFR ≥ 1). Using a 50-SNPs window (Liu *et al.* 2012), the Bayesian method reduced the noise by smoothing the peak, but the physical coordinates of linkage peaks were lost. In our method, the sliding-window played the same smoothing function and the density of SNPs was kept. Therefore, a significant gap (175–177 Mb), corresponding to the deletion of six exons within the *o1* gene, inside the BSR-seq linkage peak in mutant 937 was shown as well. This ∼1.8 Mb gap could also result from low SNP density (Figure S2C). In addition, as evidence of the relationship between SNP density and linkage peaks, the correlation of high SNP density across chromosome 4 with small linkage peaks suggested their weak linkage to the causative gene region, and this is not evident on other chromosomes. The moving steps and windows of positive SNPs/indels in BSR-seq analysis were evaluated, and for a kilobase-sized deletion, a megabase-sized window would be too large to show the linkage gap by causative deletion (Figure S6). The gap covering the causative deletion in mutant 937 was estimated to range from 1.1 Mb to 2,076,580 bp, while the gap was ∼1 Mb in mutant 916. A minimum window size of 100 kb and step size of 10 kb is needed to plot the gaps caused by causative deletion in mutants 937 and 916.

### Precise characterization of o1 deletion in mutant 937

**Figure 5:**
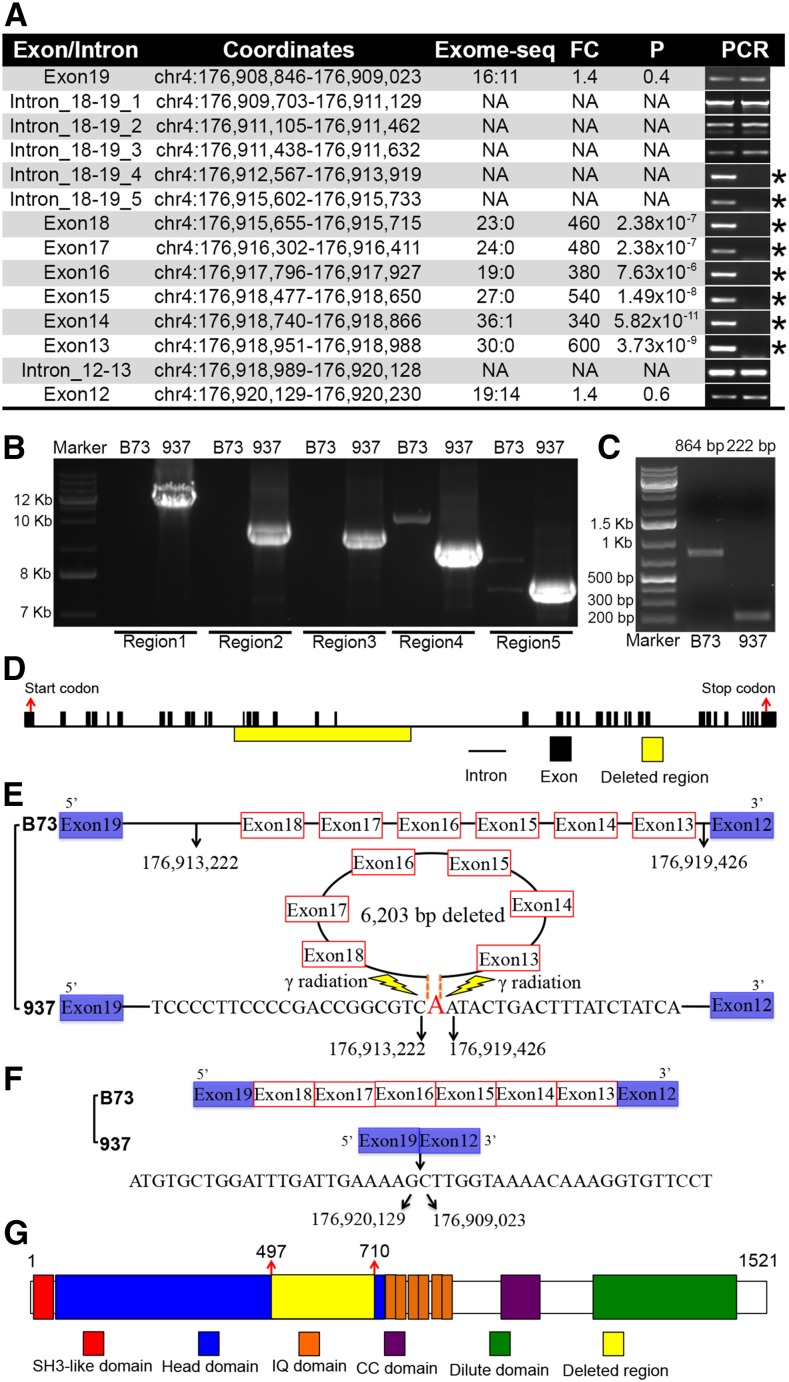
Genomic PCR and clone verification of deletion in *O1* in mutant 937. (A) Exon/Intron column is for the region where primers were designed. Mapped read depth coverage in exome-seq was normalized to compare wild-type B73 and mutant 937 (B73:937). Fold-changes (FC) of read depth coverage between B73 and mutant 937 and *P*-values were used to search for deleted exons. The verified deletions are noted with an asterisk. (B) Five long PCRs were conducted to cover the junction site, and PCR product sizes are 7584 bp, 8331 bp, 8713 bp, 9046 bp, and 11,327 bp for Region 1–5. No bands in B73 result from genomic regions too long for PCR. (C) cDNA was synthesized and RT-PCR was conducted to compare B73 and 937 and showed an ∼600 bp difference. Clone sequencing confirmed the PCR product sizes are 864 bp in B73 and 222 bp in 937. (D) The causative deletion covered six exons inside the total 39 exons of *O1* gene. (E) Clone sequencing of genomic PCR products in 937 confirmed the 6203 bp deletion inside *O1* gene and the two break points at 176,913,222 and 176,919,426 based on maize genome v3.25. (F) cDNA clone sequencing identified the transcript variant without the six deleted exons, and exons #12 and #19 were linked directly. The *O1* gene resides in the negative strand, and was shown in coordinates for the positive strand from 5′ end to 3′ end. (G) Protein domains in O1. The deletion from position 497 to 710 was in the head domain. Protein domain positions were from Wang *et al.* (2012).

To verify the deletion in mutant 937, primers were designed inside the exons and introns (Table S3), and the six deleted exons were all validated based on genomic PCR results, with two intron regions deleted as well (Figure 5, A and D). The BSR-seq and exome-seq results allowed broad definition of the deletion, and clone sequencing provided the base pair resolution. Long genomic PCR was used to cover the junction site in mutant 937, and five products were obtained by amplifying across the junction in the mutant that gives no product in wild type (Figure 5B). Sequencing of these PCR products showed the precise genomic coordinates of two break points at 176,913,222 and 176,919,426, so that the actual deletion size is 6203 bp (Figure 5E). This showed good agreement with the estimation of break points (176,912,920 and 176,919,560) in BSREx-seq analysis. It was interesting that a nucleotide residue of adenine (“A”) was left between two break points, which might be formed by DNA repair.

cDNA sequencing was used to examine *O1* gene transcript variants in mutant 937. RT-PCR identified a ∼600 bp difference between the products of wild-type B73 and mutant 937 (Figure 5C), confirming the deletion and two transcript variants. Clone sequencing showed the six deleted exons were not present in the 937 mutant transcript, and the boundaries of exons 12 and 19 (176,920,129 and 176,909,023) were linked directly (Figure 5F). The cDNA difference between wild-type and mutant 937 was the concatenated lengths of six exons (642 bp). cDNA clone sequencing showed the deletion did not change the open reading frame, and predicts a truncated O1 protein with 214 amino acids deleted out of the total length of 1521 amino acids (Figure 5G). The western blot confirmed the existence of two protein variants in normal-type and mutant 937 (Figure 4C), which confirmed the two transcript variants in BSR-seq analysis (Figure 4B). Since the deletion did not change the open reading frame, two thirds of normal kernels are expected to show the presence of both the full-length 173.4 kD protein and a detectable level of the truncated 148.9 kD protein indicating their hemizygous nature. Homozygous wild-type kernels only contain the 173.4 kD protein while homozygous mutant kernels only contain the 148.9 kD protein. The 214 deleted amino acids occur in the myosin head domain, which enables myosin to bind the filamentous actin to move inside the cell using ATP hydrolysis (Wang *et al.* 2012). Thus, the deletion likely impairs O1 function as a myosin XI motor protein (Wang *et al.* 2012) although it is an intriguing possibility that residual function remains, especially since partial complementation may be evident from the allelism test to the *o1*-reference allele (Figure 1B). The progeny kernels, while opaque, show a thin layer of vitreous endosperm.

### Limitation of BSREx-seq

Although the ability to identify functional mutations by BSREx-seq was demonstrated, mutations in extragenic regulatory regions are undetectable for the exome-seq technology. For example, mutant 883 showed very good BSR-seq linkage results on chromosomes 8, but no deleted exons or differentially expressed genes between normal and mutant types were found under the linkage peak (Figure S2D). Since γ-radiation induces random mutagenesis, and the gene-free regions are much longer than coding regions, it is expected that many deletions will be entirely within gene-free regions and that some of these will be causative. In such cases, alternative strategies must be employed to identify functional variations under the linkage peak.

## Discussion

### A novel mutational resource for functional genomics of maize seed development

Both endospermic and nonendospermic seeds develop in three overlapping phases (Egli 2006). These are a phase of cell division and minimal weight gain after fertilization, a seed filling stage when cells expand, endoreduplicate, and assimilate storage compounds and a final stage of maturation when desiccation and dormancy occur. Mutations that interrupt early stages may be more likely to cause lethality during embryogenesis or during the earliest stages of seedling growth as a result of failed or insufficient differentiation or maturation of cotyledon or endosperm tissue. Indeed, more than half of small kernel and opaque mutants identified in this population display seed or seedling lethality and as such, it is impossible to assign seed-specific functions to their causative genes. During the late seed development phase, mutants that fail to desiccate or become dormant are also often lethal and we have also identified many mutants which appear to be in this category. However, a substantial proportion of maize seed mutants in this population appear to be affected in the midstages of kernel filling, nutrient assimilation, and endosperm maturation, and often have little or no adverse effect on embryo viability or adult vegetative development. Such mutants are useful for identifying true seed-specific control factors. γ-radiation was used to create this population since it is known to create whole gene deletions, but since relatively little is known about the range of deletion sizes, its value as a functional genomics tool was not known. We not only created a unique biological resource for studying seed and whole plant development, but also showed that, although some mutations are too large to be realistically useful for functional genomics, some mutations affect single or small numbers of genes.

In an *Arabidopsis thaliana* experiment involving high-dosage γ-radiation, most of the identified deletions were large (up to 6 Mb), and the majority of these were not transmitted to progeny due to the loss of genes essential for viability (Naito *et al.* 2005). Both nucleotide substitutions and small deletions induced by γ-radiation were found in different plant species, such as *Arabidopsis* (Yoshihara *et al.* 2010), tomato (Shirasawa *et al.* 2015), and rice (Sato *et al.* 2006). In our maize mutagenesis population, only large and medium-sized deletions were confirmed or implicated as causative candidates, and no small indels and SNPs were thought to be causative. For example, three small indels and 17 SNPs with high confidence in mutant 937 were unique compared with B73 (Table S2), but none of these small variations corresponded to the BSR-seq linkage peak at 174.9 Mb of chromosome 4.

Despite the apparent extensive generation of small mutations, our confirmed or strong candidates for causative mutations were large- or medium-scale deletions, such as the 1.7 Mb deletion in mutant 1115. More importantly, a single gene deletion was confirmed as the causative mutation in mutants 937 and 146 (Table 2 and Figure 1B). This indicated that our optimized γ-radiation dosage makes a good balance between causing deletions and maintaining high-level viability. Although the deletions in 937 and 146 are still of several kilobases, the extensive number and size of introns resulted in these deletions being entirely within a single gene in both cases. In some cases where we have a precise mapping interval of linkage peak but no apparent exonic deletions in that interval such as in mutants 883 and 1554, we have to consider induced mutations within introns or in regulatory sequences.

### Improved BSR-seq not only maps the causative mutation but can highlight the deletion within the mapping peak

BSR-seq combines the convenience of bulked segregant analysis with RNA-seq using RNA extracted from pooled mutant and nonmutant plants. Furthermore, using quickly and easily obtained seedling leaf tissue RNA, causative genes can be mapped even if they are not expressed in leaf tissue, using SNPs mined from surrounding genes that are expressed (Liu *et al.* 2012, 2013). This is advantageous because seed mutant phenotypes of interest in the current collection may arise from loss of genes with seed-specific expression and the need to isolate RNA pools from developing kernels would add considerable time and expense since it requires large-scale field propagation, ear sampling, and storage. In fact, all the causative gene candidates in this study were shown (using publicly available expression data) to have high-level expression in embryo, endosperm, and whole seed (Figure S3).

### BSREx-seq for forward genetics

Whole genome scanning by exome-seq has the potential to detect the presence/absence of genes, exons, and subexon regions. However, depending on the dosage, γ-radiation mutagenesis might induce too many mutations, which could reduce its applicability for forward genetics. Using BSR-seq alone also has some limitations, and using expression difference between wild type and mutant is unlikely to unequivocally identify a causative gene. Kernel-specific, nonpleiotropic mutant phenotypes may arise from mutations in genes not expressed in leaf tissue. Furthermore, small deletions inside causative genes do not necessarily alter their expression level measured by read counts. Both of these factors make it difficult to infer the causative gene by using BSR-seq alone. Therefore, in this study, we developed BSREx-seq by combining BSR-seq and exome-seq. By scanning the mapping interval for deletions, in the small number of mutants so far studied, we have been able to confirm or strongly suggest candidates in three.

Mapping-by-sequencing is a relatively new method in forward genetic screens for identifying causative mutations behind phenotypes of interest (Schneeberger 2014). In addition, direct comparison of resequenced whole genomes can identify candidates for causative mutations (Nordström *et al.* 2013; Schierenbeck *et al.* 2015). For example, exome sequencing of phenotypic bulks identified a candidate gene in barley (Mascher *et al.* 2014), and though expensive and more labor intensive, whole genome sequencing (WGS) was able to map the causative mutation through a pooled mapping population (Blumenstiel *et al.* 2009; Schneeberger *et al.* 2009; Zuryn *et al.* 2010). BSR-seq was shown to have similar mapping intervals to WGS-based bulk segregant analysis (BSA) (Miller *et al.* 2013). With cost-effective sequencing of exon-enriched genomic DNA compared to WGS-based BSA, BSREx-seq has the ability to pool and multiplex increasingly large numbers of different mutants, and give flexibility to fit the various situations in different plant species. Combining BSR-seq mapping using the transcriptome with exome DNA sequencing represents an efficient and cost-effective approach to identify causative mutants in complex genomes.

## Supplementary Material

Supplemental Material
