# Supplementary material for: A Population of Deletion Mutants and an Integrated Mapping and Exome-seq Pipeline for Gene Discovery in Maize
Source: G3 (Bethesda). 2016 Jun 1;6(8):2385–95. doi: 10.1534/g3.116.030528 (PMC4978893; doi:10.1534/g3.116.030528)
Supplement: Supplemental Material [file supp_6_8_2385__index.html]

A Population of Deletion Mutants and an Integrated Mapping and Exome-seq Pipeline for Gene Discovery in Maize — Supplemental Material 

# A Population of Deletion Mutants and an Integrated Mapping and Exome-seq Pipeline for Gene Discovery in Maize

## Supplemental Material for Jia *et al.*, 2016

**Files in this Data Supplement:**

- Figure S1 - Combining BSR-seq and exome-seq to identify the 25-bp causative deletion in mutant 1039-*o2*. (.pdf, 1 MB)
- Figure S2 - Chromosomal plots of linkage peaks by BSR-seq and mutations by exome-seq in mutants 916, 1554, 937 and 883. (.pdf, 1 MB)
- Figure S3 - Tissue specific expression of causative gene candidates. (.pdf, 453 KB)
- Figure S4 - Exome-seq identified causative deletion candidate in mutant 146. (.pdf, 757 KB)
- Figure S5 - BSREx-seq identified a large deletion in mutant 1115. (.pdf, 1 MB)
- Figure S6 - Moving windows and steps to show linkage peaks in BSR-seq analysis. (.pdf, 1 MB)
- Table S1 - Summary of putative B73 kernel mutants. (.xlsx, 18 KB)
- Table S2 - Summary of mutants analyzed with BSR-seq and exome-seq. (.xlsx, 11 KB)
- Table S3 - Primers for genomic PCR and RT-PCR in mutants 937 and 916. (.xlsx, 173 KB)
- Table S4 - Kernel number in F2 generation and Chi-square test for Mendelian inheritance. (.xlsx, 10 KB)
- Table S5 - Causative deleted gene candidates in mutant 1115. (.xlsx, 18 KB)
- Table S6 - Assembled transcript variants in the *O1* gene of 937 mutant. (.xlsx, 11 KB)
