# Supplementary material for: A Population of Deletion Mutants and an Integrated Mapping and Exome-seq Pipeline for Gene Discovery in Maize
Source: G3 (Bethesda). 2016 Jun 1;6(8):2385–95. doi: 10.1534/g3.116.030528 (PMC4978893; doi:10.1534/g3.116.030528)
Supplement: Supplemental Material [file supp_g3.116.030528_FigureS1.pdf]

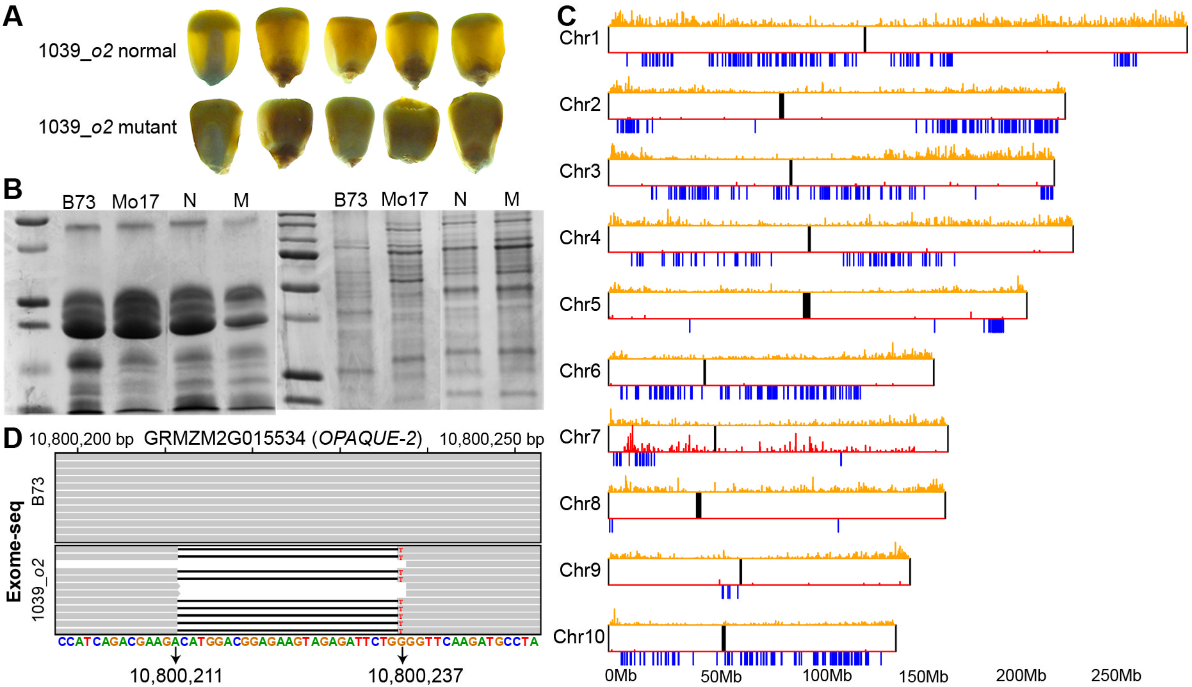

Figure S1 Combining BSR-seq and exome-seq to identify the 25-bp causative deletion in mutant 1039-*o2*. (A) Full size vitreous/opaque kernels in 1039-*o2* x Mo17 F2 segregating ears were imaged on a light box. (B) SDS-PAGE for zein (left) and non-zein (right) in a comparison of normal type (N) and mutant type (M). (C) Chromosomal plot of linkage peaks from BSR-seq and exon-deletions by exome-seq. Orange bars: SNP/indel density; red bars: numbers of positive SNPs/indels in sliding-windows of 100 Kb with a step of 10 Kb; blue bars: unique deleted exons by detected exome-seq; one brown bar: the causative deletion in o2 gene; black bars: the physical positions of centromeres (Wolfgruber et al. 2009). Y axis scale for positive SNPs/indels ranges from 0 to 16. (D) The causative deletion (black line), in a comparison of B73 and mutant 1039-*o2*, is 25-bp length with a T allele on the right end, known as o2T. The genomic coordinates were shown within exon #4 of O2 gene in exome-seq. The consensus sequences are shown in the bottom. Grey bars are for the mapped reads.

Wolfgruber, T.K., A. Sharma, K.L. Schneider, P.S. Albert, D.H. Koo et al., 2009 Maize centromere structure and evolution: sequence analysis of centromeres 2 and 5 reveals dynamic loci shaped primarily by retrotransposons. PLoS Genet. 5: e1000743.
