# Supplementary material for: A Population of Deletion Mutants and an Integrated Mapping and Exome-seq Pipeline for Gene Discovery in Maize
Source: G3 (Bethesda). 2016 Jun 1;6(8):2385–95. doi: 10.1534/g3.116.030528 (PMC4978893; doi:10.1534/g3.116.030528)
Supplement: Supplemental Material [file supp_g3.116.030528_FigureS2.pdf]

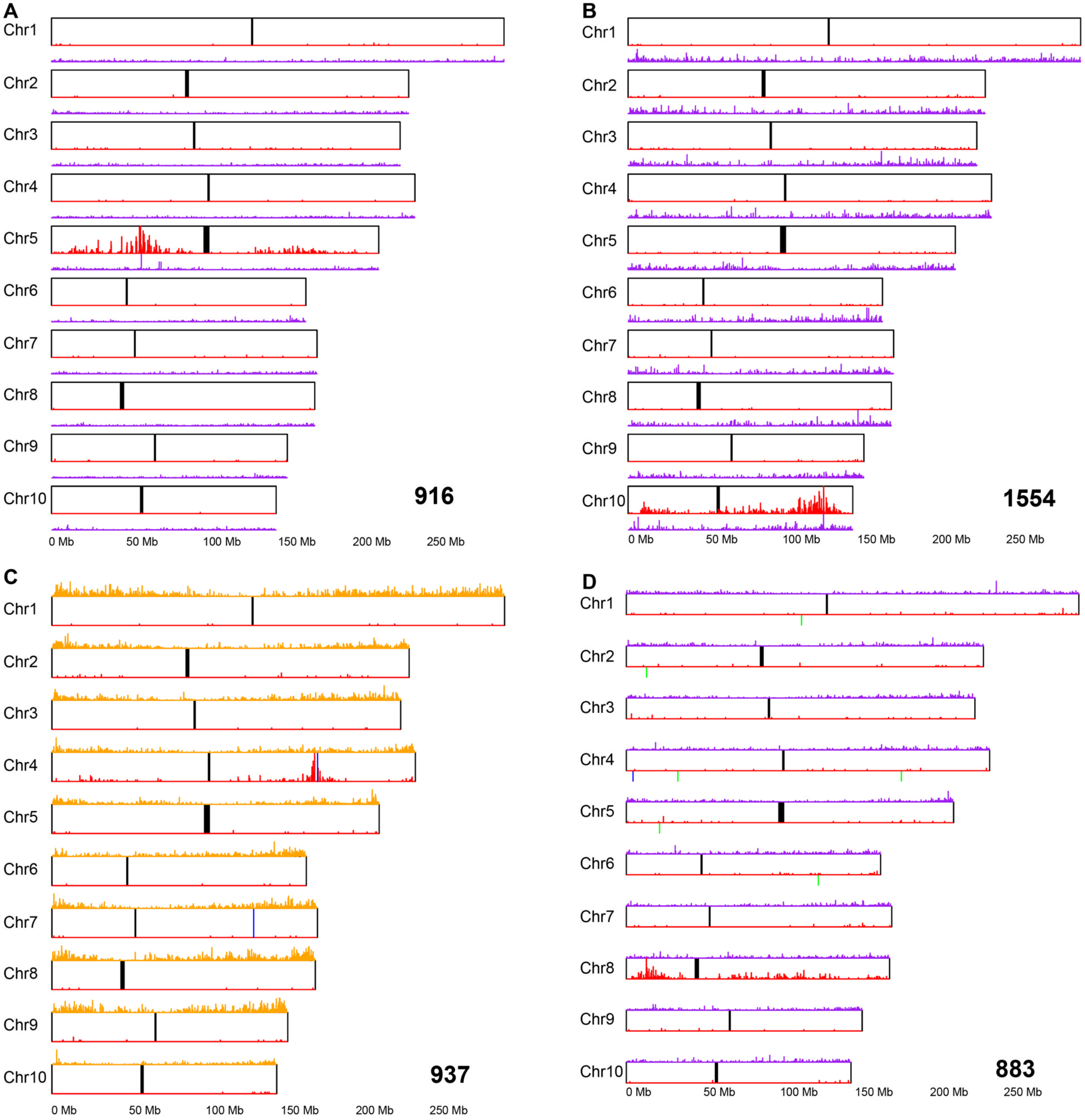

Figure S2 Chromosomal plots of linkage peaks by BSR-seq and mutations by exome-seq in mutants 916, 1554, 937 and 883. All ten chromosomes are shown for mutants 916 (A), 1554 (B), 937 (C), and 883 (D), with linkage peaks and expression fold-change by BSR-seq, and mutations by exome-seq. Red, linkage curve by positive SNP/indel density value in a sliding window of 100 kb with a step of 10 kb; black, centromeres; blue, unique deleted exons by exome-seq; green, unique small indels; purple, gene expression fold-change between normal and mutant; orange, SNP/indel density. Y axis scale for positive SNPs/indels ranges from 0 to 23 (mutant 916), 31 (mutant 1554), 20 (mutant 937), and 17 (mutant 883).
