# Supplementary material for: A Population of Deletion Mutants and an Integrated Mapping and Exome-seq Pipeline for Gene Discovery in Maize
Source: G3 (Bethesda). 2016 Jun 1;6(8):2385–95. doi: 10.1534/g3.116.030528 (PMC4978893; doi:10.1534/g3.116.030528)
Supplement: Supplemental Material [file supp_g3.116.030528_FigureS3.pdf]

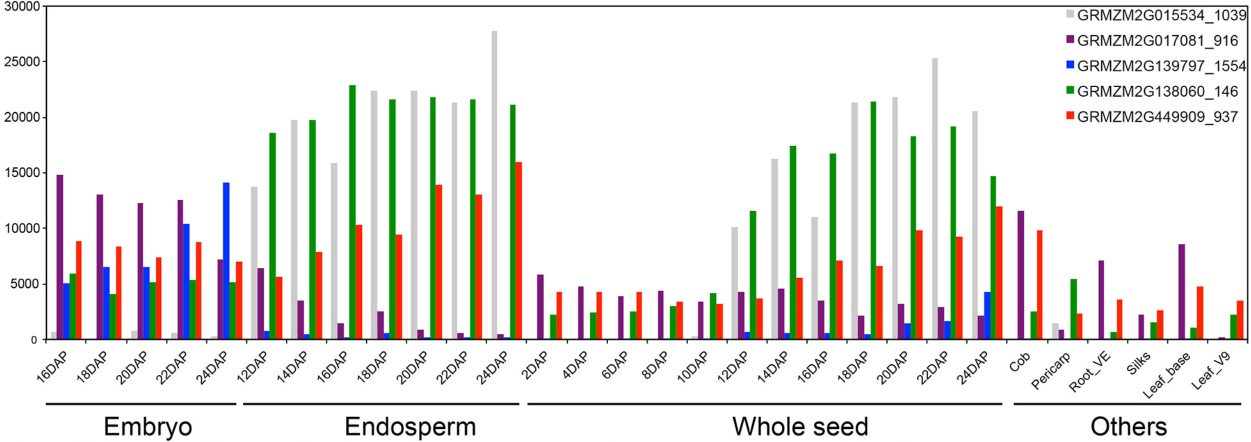

Figure S3 Tissue specific expression of causative gene candidates. The gene expression was retrieved from the genome-wide tissue transcription by Sekhon et al. (Sekhon et al. 2011). Cob, immature cob at V18; Pericarp, pericarp at 18DAP; Root_VE, primary root at VE; Silks, silks at R1; Leaf_base, leaf at base of stage-2; Leaf_V9, eleventh leaf at V9.

Sekhon, R.S., H. Lin, K.L. Childs, C.N. Hansey, C.R. Buell et al., 2011 Genome-wide atlas of transcription during maize development. Plant J. 66: 553-563.
