# Supplementary material for: A Population of Deletion Mutants and an Integrated Mapping and Exome-seq Pipeline for Gene Discovery in Maize
Source: G3 (Bethesda). 2016 Jun 1;6(8):2385–95. doi: 10.1534/g3.116.030528 (PMC4978893; doi:10.1534/g3.116.030528)
Supplement: Supplemental Material [file supp_g3.116.030528_FigureS4.pdf]

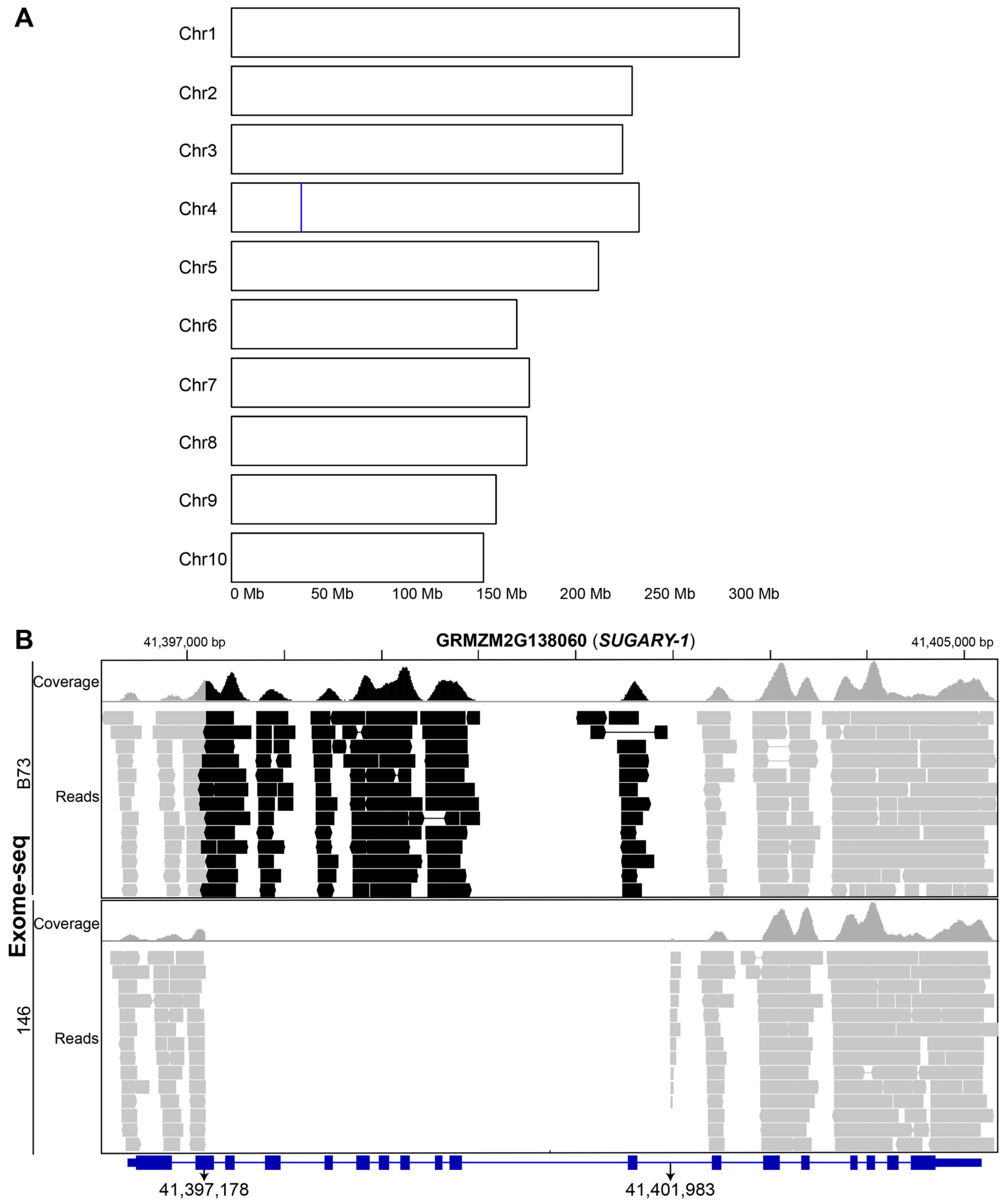

Figure S4 Exome-seq identified causative deletion candidate in mutant 146. (A) Chromosomal plot of the unique deletion by exome-seq. Only one unique exon-deleted region (blue) was identified in mutant 146. (B) ~4.8 kb DNA fragment (black, chr4:41,401,983-41,397,178) deleted by γ-radiation, was shown in the windows of coverage and read in exome-seq. Ten exons, referring to exons #2-#11, were lost within the SUGARY-1 gene (GRMZM2G138060, totally 18 exons), and exon #2 was partially deleted. The two break points were estimated at 41,397,178 and 41,401,983 based on maize genome v3.25.
