# Supplementary material for: A Population of Deletion Mutants and an Integrated Mapping and Exome-seq Pipeline for Gene Discovery in Maize
Source: G3 (Bethesda). 2016 Jun 1;6(8):2385–95. doi: 10.1534/g3.116.030528 (PMC4978893; doi:10.1534/g3.116.030528)
Supplement: Supplemental Material [file supp_g3.116.030528_FigureS5.pdf]

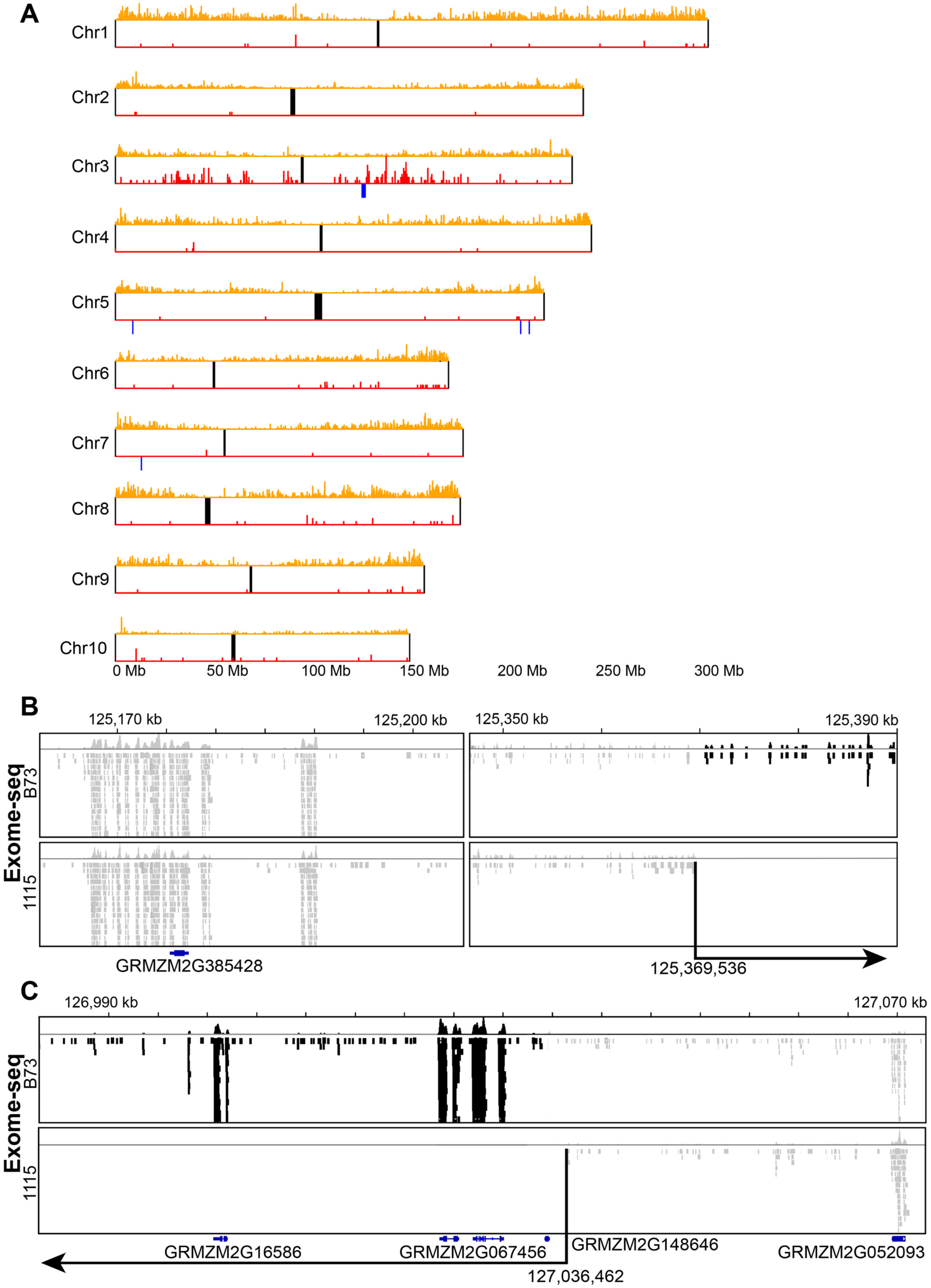

Figure S5 BSREx-seq identified a large deletion in mutant 1115. (A) Chromosomal plotting of linkage peaks by BSR-seq and deletions by exome-seq. Red bars: numbers of positive SNP/indel in sliding-windoww of 100 kb with a step of 10 kb; blue bar: unique deleted exons by exome-seq; black bar: the physical positions of centromere; orange, SNP/indel density in a sliding window of 100 kb with a step of 10 kb. The left break point (B) at genomic position of 125,369,536, and the right break point (C) at 127,036,462 were estimated based on the mapped reads in the coverage and read windows of IGV in exome-seq.
