# Supplementary material for: A Population of Deletion Mutants and an Integrated Mapping and Exome-seq Pipeline for Gene Discovery in Maize
Source: G3 (Bethesda). 2016 Jun 1;6(8):2385–95. doi: 10.1534/g3.116.030528 (PMC4978893; doi:10.1534/g3.116.030528)
Supplement: Supplemental Material [file supp_g3.116.030528_FigureS6.pdf]

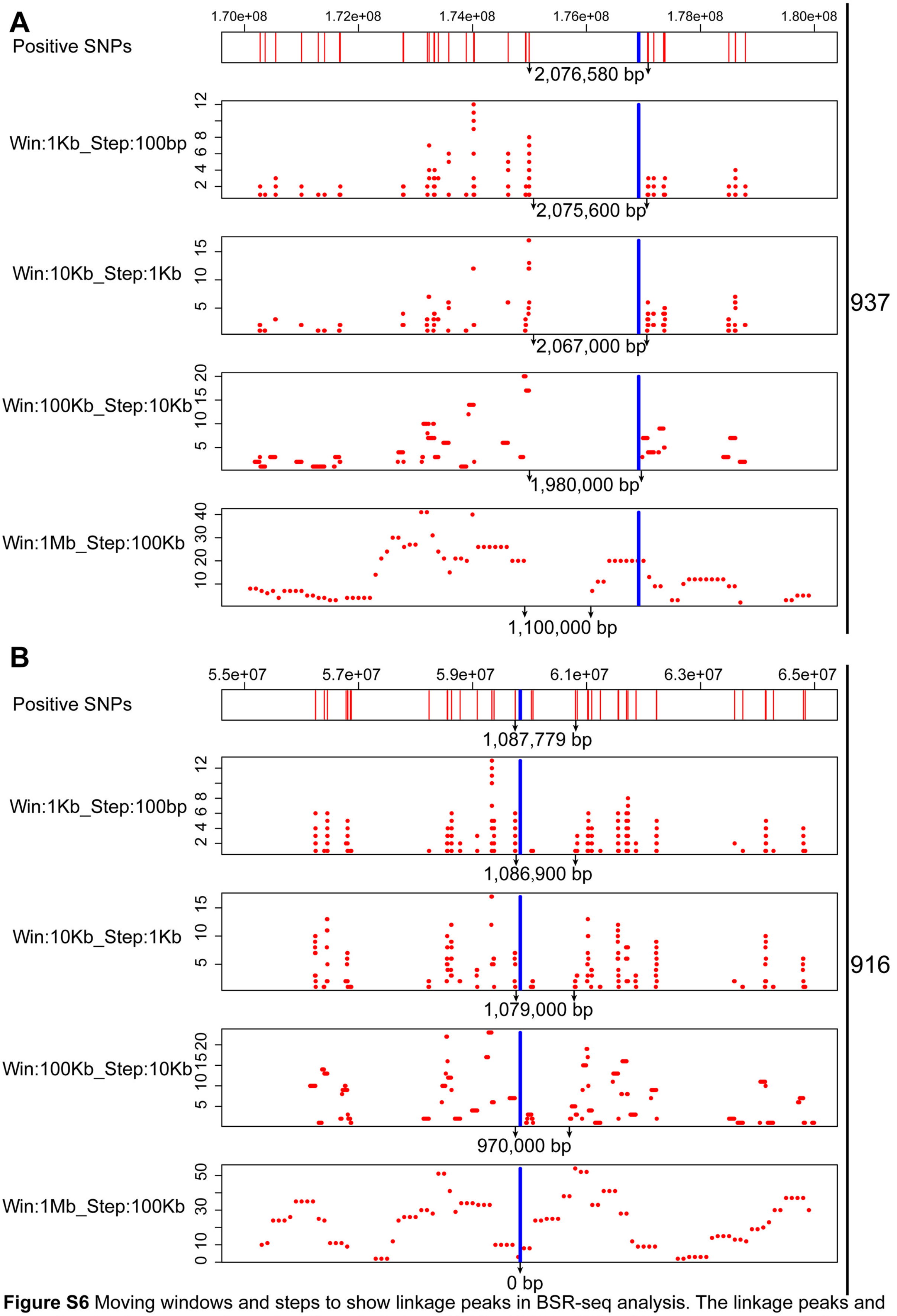

Figure S6 Moving windows and steps to show linkage peaks in BSR-seq analysis. The linkage peaks and causative deletions were shown in different windows (1 Mb, 100 kb, 10 kb, 1 kb and presence of all positive SNPs/indels) and steps (100 kb, 10 kb, 1 kb, 100 bp and presence of all positive SNPs/indels), and the lengths of gaps covering causative deletion were shown to increase with smaller window and step. It showed that at least a window size of 100 kb and step size of 10 kb is needed to plot the gaps caused by causative deletion in mutants 937 (A) and 916 (B).
